# PREDICT Tool for Pregnancy-Associated CKD Progression

**DOI:** 10.1016/j.ekir.2026.106636

**Published:** 2026-06-06

**Authors:** Elizabeth Ralston, Mairéad Hamill, Shalini Santhakumaran, Michelle Hladunewich, Graham Smith, Lavanya Bathini, Nivethika Jeyakumar, Amit X. Garg, Kate Bramham, PREDICT Investigation Group, Elizabeth Ralston, Kate Wiles, Michelle Hladunewich, Yanzhong Wang, Amanda Clery, Joseph Chilcot, Chris Farmer, Steve Childs, Yuanhang Yang, Nivethika Jeyakumar, Amit Garg, Lavanya Bathini, Graham Smith, Hannah Blakey, Nadia Sarween, Graham Lipkin, Ellen Knox, Tess Harris, David Pitcher, Shalini Santhakumaran, Anna Casula, Retha Steenkamp, Lucy Chappell, Philip Webster, Sue Carr, Matthew Hall, Liz Lightstone, Kate Bramham, RaDaR Consortium

**Affiliations:** 1 Department of Women and Children’s Health, Faculty of Life Sciences and Medicine, School of Life Course and Population Sciences, King’s College London, London, UK; 2 UK Renal Registry, UK Kidney Association, Bristol, UK; 3 Division of Nephrology, Sunnybrook Health Sciences Centre, Temerty Faculty of Medicine, University of Toronto, Toronto, Ontario, Canada; 4 Institute for Clinical Evaluative Sciences (ICES), Toronto, Ontario, Canada; 5 London Health Sciences Centre Research Institute, London, Ontario, Canada; 6 Department of Medicine, Schulich School of Medicine and Dentistry, Western University, London, Ontario, Canada; 7 Department of Epidemiology and Biostatistics, Schulich School of Medicine and Dentistry, Western University, London, Ontario, Canada

**Keywords:** chronic kidney disease, obstetric nephrology, prediction tool

## Abstract

**Introduction:**

Prepregnancy counselling is recommended for women with chronic kidney disease (CKD) to discuss potential adverse outcomes; however, no tools exist to estimate individual risk. We aimed to develop and externally validate 2 prediction models for outcomes prioritized by people with CKD and health care professionals: The primary outcome was the probability of ≥25% reduction in estimated glomerular filtration rate (eGFR) or kidney replacement therapy (KRT) within 12 months postpartum. The secondary outcome was the probability of small-for-gestational-age (SGA) (< 3rd percentile) infant and/or preterm delivery (< 34 weeks).

**Methods:**

The development cohort used linked data from the National Registry of Rare Kidney Disease (RaDar), UK Renal Registry (UKRR) and NHS Hospital Episode Statistics (HES). Individuals with eGFR < 90 ml/min per 1.73 m^2^ within 24 months preconception and deliveries between 1997 and 2021 were included. Validation cohorts were as follows: (i) Ontario Pregnancy Cohort (2007–2022) and (ii) 3 UK pregnancy-CKD studies (2010–2018). Candidate predictors were selected from known risk factors. Clinically relevant cut-points were determined with people with CKD.

**Results:**

The development cohort included 746 women (median prepregnancy eGFR: 58 ml/min per 1.73 m^2^); validation cohorts included 6974 and 380 women. Optimal cut-points of 0.15 (sensitivity: 90%, negative predictive value (NPV): 85%) and 0.10 (sensitivity: 90%, NPV: 80%) were selected for the primary and secondary outcomes. External validations demonstrated high sensitivity and NPV for the primary outcome. Although comparability is limited by differing end points, Kidney Failure Risk Equation (KFRE) predictions (2-year) were lower than our PREDICT model (1-year) (median risk 1.4% vs. 48%).

**Conclusion:**

We developed high performing models for individuals with CKD to predict coselected adverse kidney and neonatal outcomes from contemporaneous cohorts. Individualized pregnancy risk assessment tools could support future parents and health care professionals to make informed choices.

## Graphical abstract

**Figure:**
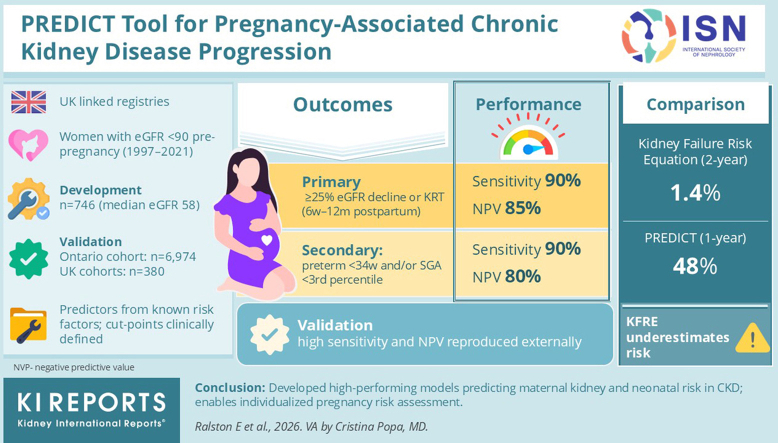

CKD stages 1 to 5 is estimated to affect ≤6% and 12% of women of reproductive age in high- and low-income countries, respectively, and the number of those affected is projected to increase.^1^, ^2^, ^3^ Prepregnancy counselling is recommended for all people with CKD to discuss the risk of accelerated decline in kidney function and other adverse maternal and neonatal outcomes, including preeclampsia, SGA infants, and preterm birth.^4^, ^5^, ^6^

Individuals contemplating pregnancy, especially those with advanced CKD, may be faced with the conflicting desire for parenthood and need to balance the strain of progression to KRT and potential life-long impact of preterm birth. Clinicians are required to provide accurate communication of risk and holistic pregnancy planning, including optimal timing of conception, frequency and nature of surveillance, timing of delivery, and management postpartum. Although pregnancies are more complicated for women with CKD than in healthy pregnancy, many women have successful outcomes, and very few require KRT during or soon after pregnancy. However, many clinicians remain reluctant to support pregnancy in individuals with advanced CKD. Some patients may choose not to conceive, and others remain anxious throughout pregnancy because of perceived risk of adverse outcomes.

Currently no prediction tool exists that accurately estimates the risk of kidney disease progression following pregnancy, or the risk of preterm birth or SGA infant. A reliable prediction tool would guide future parents and their health care professionals to provide reassurance for those likely to have good outcomes, inform optimal timing of pregnancy, and support risk stratification and targeted interventions for those with the greatest need, including expedited education regarding dialysis modalities, and preliminary transplant work-up.

We aimed to develop and externally validate 2 prediction models, which estimate primary and secondary outcomes coselected by people with CKD and health care professionals: the primary outcome is the probability of ≥ 25% reduction in eGFR or KRT within 12 months postpartum; the secondary outcome is the probability of SGA (< 3^rd^ percentile) infant and/or preterm delivery (< 34 weeks’ gestation).

## Methods

### Study Population

#### Development Cohort

The development cohort was derived from linkage between:

1. RaDaR, which contains automatic feeds of laboratory data for consented patients in a number of rare disease groups;
2. UKRR, a UK-wide registry of validated clinical and demographic data on all patients on KRT and 42% of centers submitting data on people with advanced CKD; and
3. HES, a repository of data on inpatient and outpatient activity from all NHS trusts in England, including the International Classification of Diseases, 10th Revision diagnostic codes.

This cohort included individuals with a delivery in HES between April 1997 and March 2021 with an eGFR < 90 ml/min per 1.73 m^2^ recorded in RaDaR/UKRR data within 24 months before conception. Those established on dialysis at the time of conception, known inpatient eGFR measurement or with no preconception eGFR within 24 months or postpartum eGFR within 6 weeks to 12 months were excluded. Follow-up data were available until December 2021 for the UKRR and September 2023 for RaDaR. Patients who have opted out of the use of their data for research via the National Data Opt-out were not included.

#### Validation Cohorts

External validation was performed using the following datasets:

1. The Ontario Pregnancy Cohort, a population-based cohort of women in the province of Ontario, Canada with a delivery between April 2007 and March 2022. Administrative health databases linked using unique encoded identifiers at ICES were used to capture all hospital births in Ontario and outpatient laboratory testing. ICES is an independent, nonprofit research institute whose legal status under Ontario’s health information privacy law allows it to collect and analyze health care and demographic data, without consent, for health system evaluation and improvement.
2. Cohort data from 3 UK prospective cohort studies of pregnant people with CKD between 2010 and 2018, which have previously been described in detail.^5^^,^^7^^,^^8^

The selection of cohorts for development and validation was determined by timing of data access and resources available.

### Ethical Approval

Ethical approval was given by the Regional Ethics Committee and Health Research Authority (London Bloomsbury Research Ethics Committee, Ref: 23/LO/0258). The use of the Ontario data in this project was authorized under section 45 of Ontario’s Personal Health Information Protection Act and did not require review by a Research Ethics Board. The study was registered on ClinicalTrials.gov (Ref:NCT05793346). This study involved human participants and was approved by the UKRR, which holds permissions under s251 of the National Health Service (NHS) Act 2006 to gather, process, and share confidential patient information for the purposes of audit and research. These permissions are renewed annually by the UK’s Health Research Authority’s Confidentiality Advisory Group. The collection and analysis of the data for research were carried out under the ethical permissions granted to the UKRR by the Research Ethics Committee (Ref: 16/NE/0042). This study was approved by the UKRR Data Release Group. All internal and external applications for UKRR data were reviewed and approved by members of the UK Kidney Association Patient Council. Research outputs were disseminated via the UK Kidney Association website, where a lay summary is provided.

### Outcomes

Outcomes were chosen from self- reported questionnaires, developed using a modified version of the Perception of Pregnancy Risk Questionnaire, including 90 women with CKD and 73 health care professionals.^9^ The selected primary outcome was a ≥25% reduction in median eGFR from prepregnancy values or initiation of KRT between 6 weeks and 12 months postpartum. This timeframe was chosen because of serum creatinine concentrations stabilizing after peripartum deterioration^10^ and pregnancy unlikely to be a contributing factor to progression beyond this period. eGFR was calculated for the UK cohorts using the CKD Epidemiology Collaboration 2009^11^ without ethnicity adjustment, as recommended for clinical care in UK, and for the Ontario cohort using the CKD Epidemiology Collaboration 2021 equation without ethnicity adjustment. An index date (taken as the estimated conception date for those with gestational age data and 42 weeks before delivery otherwise) was assigned to each woman and prepregnancy characteristics and median creatinine were assessed in the 24 months before the index date. Inpatient assessments of kidney function were excluded because of potential concurrent acute kidney injury. The secondary outcome was a composite outcome of preterm birth defined as <34 weeks’ gestation and/or SGA < third percentile calculated using INTERGROWTH-21 centiles.^12^

### Predictor Variables

Candidate model predictors were selected from previous studies and meta-analyses reporting routinely collected risk factors for pregnancy and kidney outcomes in women with CKD and proportion of missing data within the cohorts (Tables 1 and 2) to facilitate future implementation. Proteinuria within 2 years of the index date was defined as normal (urine albumin-to-creatinine ratio (uACR) < 3 mg/mmol or urine protein-to-creatinine ratio (uPCR) < 15 mg/mmol), moderate (uACR: 3–30 mg/mmol, uPCR: 15–50 mg/mmol) or severe (uACR > 30 mg/mmol, uPCR > 50 mg/mmol). uACR was used in preference to uPCR and the most recent measurement before pregnancy included. Hypertension was determined using the HES International Classification of Diseases, 10th Revision, or if the woman had hypertension recorded as the primary cause of CKD. Diabetes was determined using the HES International Classification of Diseases, 10th Revision codes, or if the woman had diabetes mellitus recorded as the primary cause of CKD, excluding gestational diabetes. Diabetes and hypertension were recorded in the UK validation prospective cohorts, and in Ontario were assessed using standardized database codes.

**Table 1:** Baseline characteristics and outcomes of the development and validation cohorts

Characteristics		Development cohort (*n* = 746)	Validation cohort (Ontario, Canada) (*n* = 6974)	Validation cohort (UK) (*n* = 380)
Baseline characteristics				
Maternal age at conception, yrs		32 (29–36)	34 (30–37)	32 (27–37)
Maternal ethnicity^a^	Asian	93 (13%)	n/a	59 (16%)
	Black	30 (4%)	n/a	74 (20%)
	Mixed/Other	22 (3%)	n/a	26 (7%)
	White	597 (81%)	n/a	219 (58%)
Maternal BMI at first pregnancy visit^a^		26 (23–30)	n/a	27 (21–33)
Primary kidney disease^a^	Glomerulonephritis	144 (20%)	n/a	68 (18%)
	Chronic pyelonephritis or vesicoureteral reflux	64 (9%)	28 (0%)	54 (14%)
	ADPKD	85 (12%)	660 (10%)	26 (7%)
	Diabetic nephropathy	11 (2%)	n/a	21 (6%)
	Congenital/inherited	22 (3%)	n/a	21 (6%)
	Transplant	350 (49%)	57 (1%)	87 (23%)
	Lupus	10 (1%)	32 (1%)	21 (6%)
	Other	35 (5%)	n/a	75 (20%)
Nulliparity^a^, *n* (%)		337 (46%)	3003 (43%)	161 (42%)
Prepregnancy diabetes, *n* (%)		70 (9%)	177 (3%)	11 (3%)
Hypertension, *n* (%)		447 (60%)	412 (6%)	207 (55%)
Preconception median eGFR, ml/min per 1.73 m^2^		58 (44–72)	84 (79–87)	53 (41–64)
Proteinuria^a^	Normal	150 (41%)	6075 (87%)	61 (16%)
	Moderate	103 (28%)	508 (7%)	68 (18%)
	Severe	117 (32%)	391 (6%)	114 (30%)

ADPKD, autosomal dominant polycystic kidney disease; BMI, body mass index; eGFR, estimated glomerular filtration rate; PKD, polycystic kidney disease.

a Missing data: development cohort: ethnicity, *n* = 4; BMI, *n* = 571; PKD, *n* = 25; nulliparity, *n* = 8; proteinuria, *n* = 376. Ontario validation cohort: primary kidney disease, *n* = 6197, maternal BMI. UK validation cohort: ethnicity, *n* = 2; nulliparity, *n* = 23; prepregnancy diabetes, *n* =255; hypertension, *n* = 52; proteinuria, *n* = 137.

**Table 2:** Outcomes of the development and validation cohorts

Outcome		Development cohort (*n* = 746)	Validation cohort (Ontario, Canada) (*n* = 6974)	Validation cohort (UK) (*n* = 380)
Primary outcome				
≥ 25% decline in baseline eGFR or KRT		221 (30%)	201 (3%)	74 (20%)
Secondary outcome				
Preterm (34 wks) or SGA 3rd centile^a^		151 (26%)	494 (7%)	91 (24%)
Kidney and pregnancy outcomes				
Dialysis (within 12 mos postpartum)		46 (6%)	24 (0%)	10 (2.6%)
Transplantation (within 12 mos postpartum)		6 (1%)	n/a	n/a
Live birth^a^, *n* (%)		628 (99%)	6963 (100%)	361 (95%)
Mode of delivery^a^, *n* (%)				
	Vaginal delivery	233 (31%)	4148 (60%)	126 (33%)
	Elective cesarean	209 (28%)	2795 (40%)	107 (28%)
	Emergency cesarean	238 (32%)		99 (26%)
	Forceps/vacuum/breech	66 (9%)	533 (8%)	20 (5%)
	Unknown	0 (0%)	31 (0%)	24 (6%)
Gestational age (wks), median (IQR)		37 (34–38)	39 (37–39)	37 (34–38)
Gestational diabetes, *n* (%)		47 (6%)	128 (2%)	n/a
Birthweight (g), median (IQR)		2640 (2060–3100)	3300 (2930–3650)	2580 (2040–3010)
Birthweight centile, median (IQR)		42 (20–68)	62 (39–84)	35 (14–58)

eGFR, estimated glomerular filtration rate; KRT, kidney replacement therapy; IQR, interquartile range; SGA, small-for-gestational-age.

a Missing data: Development cohort: secondary outcome, *n* = 173; live birth, *n* = 113; birthweight, *n* = 115; gestational age, *n* = 168. Ontario validation cohort: transplantation within 12 mos and type of cesarean delivery not available. UK validation cohort: live birth, *n* = 9; mode of delivery, *n* = 24.

### Sample Size

This was a secondary analysis of preexisting data and therefore the sample size was fixed. The full model included 4 predictors, with 5 predictor parameters, obtaining 44.2 events/parameter. A formal sample size calculation was performed using the pmsampsize package,^13^ assuming an anticipated Nagelkerke *R*^*2*^ of 0.15 and an outcome prevalence of 0.46 based on a contemporaneous cohort study^5^; the minimum required sample size was 382 pregnancies, corresponding to 176 outcome events.

### Model Development

Model development and validation followed the “Transparent Reporting of a multivariable prediction model for Individual Prognosis Or Diagnosis” (i.e., TRIPOD) statement recommendations.^14^ Development of both models followed the same procedure because both outcomes are dichotomous. Initial univariable logistic regression models were performed on each candidate predictor to initially assess their crude association with the outcome, including checking departures from linearity. Linearity was assessed using locally estimated scatterplot smoothing plots and by comparing models with and without nonlinear terms using likelihood ratio tests. A multivariable model was created containing all predictors. Then backwards elimination was used to successively remove the least significant predictors in the model. A liberal *P* value of 0.10 was applied for retention. Variance inflation factors were calculated before fitting the final model to identify any collinearity between the predictor variables. The final predictors were included in a multivariable logistic regression model.

### Missing Data

Missing data were described for all candidate predictors. Where possible, multiple imputation with chained equations was used to impute missing covariate data. If missing data were substantial, this was presented as the main model to reduce selection bias and increase sample size, with the complete case model as a sensitivity analysis. Two hundred imputations were performed using predictive mean matching for continuous variables and logistic regression for categorical variables. uACR and uPCR were imputed as continuous variables before deriving categories. The imputed datasets were appended, and variable selection was performed, weighting the observations using the inverse of the number of imputations to correct the standard errors.^15^ Once the variables were selected, a logistic regression model including these variables was run on each imputed dataset separately, and the parameter estimates of interest combined using Rubin’s rules.

### Model Performance

Final models were assessed for overall performance (model fit), calibration and discrimination using the area under the receiver operating characteristic curve, Nagelkerke *R*^*2*^, Brier score, and calibration intercept and slope. Clinical performance was assessed through positive predictive value (PPV), NPV, sensitivity, and specificity. Model performance was assessed both overall and in predefined subgroups (eGFR < 60 ml/min per 1.73 m^2^, eGFR < 45 ml/min per 1.73 m^2^, transplant). A range of cut points were considered for primary and secondary outcomes with comparison of clinical performance metrics and discrimination. Stakeholders (women with CKD, nephrologists, obstetricians, obstetric physicians, midwives) selected final cut points based on prioritization of high sensitivity and NPV as the most clinically valuable approach to minimize false negatives; that is, incorrectly assigning individuals to be at low risk of adverse outcomes. As part of this process, a selection of cut points were discussed with 5 women with a range of kidney function who had recently had pregnancies, including 2 with adverse outcomes. Details of how the model could perform were explained using concepts from the Shiny tool by RStudio (NHS Tools, 2025). This open-source R package, recommended by the NHS, enables the creation of interactive web applications that can be used to communicate personalized risk. Women preferred to have intensive monitoring to facilitate timely delivery to protect kidney function if they were considered to be high-risk, even though most would have an uncomplicated pregnancy, and were only content to have low-risk care if there was a high certainty they would not have complications.

### Model Validation

The final models were externally validated to assess performance using national and international cohorts. The overall predictive performance, clinical performance, discrimination, and calibration were evaluated in each cohort. Comparison with KFRE was undertaken for hypothetical cases, and for those who had the primary outcomes in the development cohort.

All analyses were conducted using SAS version 9.4 (SAS Institute, Cary, NC).

## Results

The development and 2 validation cohorts included 746, 6974, and 380 women, respectively, with data on the primary outcome. Five hundred seventy-three of the development cohort, as well as 6974 and 359 of the validation cohorts, had data on the secondary outcome. The median age of the development cohort was 32 (interquartile range [IQR]: 29–36) years, that of the Ontario validation cohort was 34 (IQR: 30–37) years, and that of the UK validation cohort was 32 (IQR: 27–37) years. The baseline characteristics of these cohorts are presented in Table 1. Sex was defined as biological sex recorded in clinical records; gender was defined as self-reported gender identity; all participants were female sex and identified as women. The primary outcome occurred in 221 (29.6%), 201 (2.9%), and 74 (19.5%) of the development and 2 validation cohorts and secondary outcome occurred in 151 (26.4%), 494 (7.1%), and 91 (23.9%), respectively.

**Figure 1:**
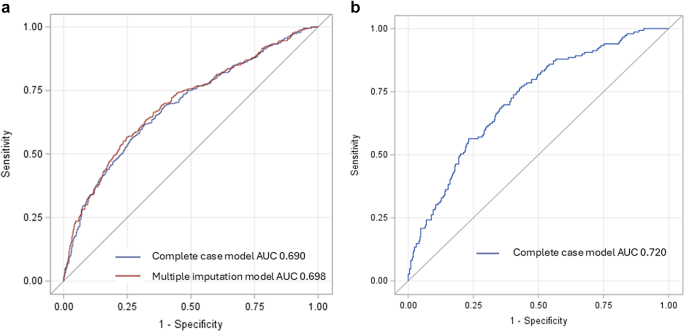
Receiver operating characteristic curve for (a) primary outcome and (b) secondary outcome.∗. ∗Multiple imputation was not performed for the secondary outcome because of the low levels of missing data. AUC, area under the curve.

Data were missing for 5 candidate predictors, including maternal body mass index (BMI) for 571 (77%) women in the development cohort. Because there was no evidence of an association with the primary or secondary outcome among the women with data, we excluded this as a candidate variable. The remaining predictors with missing data (Table 1, Supplementary Table S1) were imputed using multiple imputation as described above. The resulting model for the primary outcome had the following 4 predictors: maternal age, kidney transplant, preconception eGFR, and proteinuria (Supplementary Table S2). For the secondary outcome, only 4 additional observations could be used; therefore, a complete case analysis on 567 women was performed. The variables selected were maternal age, transplant, preconception eGFR, diabetes, and parity (Supplementary Table S2). Model performance measures for the development cohort and subgroups, for primary and secondary outcomes, are shown in Table 3 and receiver operating characteristic curve in Figure 1a and b. For the primary outcome, the model performance was similar in the multiple imputation and complete case models.

**Table 3:** Performance of models for primary and secondary outcomes

Cut point	Sensitivity	Specificity	PPV	NPV
Model for primary outcome for various cut points				
0.21	0.80	0.41	0.36	0.83
0.18	0.85	0.32	0.35	0.84
0.15	0.90	0.23	0.33	0.85
0.13	0.95	0.12	0.31	0.85
0.08	1.00	0.01	0.30	1.00
Validation in Canadian cohort				
0.15	0.82	0.38	0.04	0.99
Validation in UK external cohort				
0.15	0.96	0.08	0.26	0.85
Model for secondary outcome for various cut points				
0.15	0.80	0.31	0.33	0.78
0.14	0.85	0.28	0.34	0.81
0.10	0.90	0.17	0.32	0.80
0.08	0.95	0.10	0.31	0.82
0.06	1.00	0.04	0.31	1.00
Validation in Ontario cohort				
0.10	1.00	0.01	0.10	0.9

NPV, negative predictive value; PPV, positive predictive value.

Validation in UK external cohort not performed for secondary outcome owing to low case rate.

For the models for the primary and secondary outcomes, a range of potential cut points with a comparison of clinical performance metrics and discrimination are presented in Table 3 and receiver operating characteristic curve in Figure 1a and b. For the primary outcome, a cut point of 0.15, which has favorable clinical performance metrics with high sensitivity (90%) and NPV (85%) but lower specificity (23%) and PPV (33%). Validation in the Ontario cohort with this cut point had lower sensitivity of 82% but NPV of 99% remained high, with low specificity of 38% and PPV of 4%. Validation in the independent UK external cohort with this cut point had a sensitivity of 96% and NPV of 85% with low specificity of 8% and PPV of 26% (Table 3). For the secondary outcome, a cut point of 0.10, which has favorable clinical performance metrics with high sensitivity (90%) and NPV (80%) but lower specificity (17%) and PPV (32%) was selected. Validation in the Ontario cohort had high sensitivity and NPV (Table 3). Validation of the secondary outcome was not performed in UK external cohort due to low case rate.

Risk prediction using the PREDICT models for primary and secondary outcomes with comparisons with KFRE are shown in Table 4. Women in the development cohort who started dialysis or underwent transplantation within 2 years postpartum (i.e., KFRE outcome), who had complete data before pregnancy (*n* = 25) had a median risk estimated by the 2-year KFRE of 1.4% (IQR: 0.4%–6.1%) compared with the PREDICT model for primary outcome (KRT within 12 months) of 48% (IQR: 33.0%–55.5%).

**Table 4:** Predicted probability of outcomes for hypothetical patient profiles

Patient A		Patient B	
35-yr-old kidney transplant recipient with preconception eGFR of 40 ml/min per 1.73 m^2^ and uACR of 20 mg/mmol		35-yr-old with preconception eGFR of 40 ml/min per 1.73 m^2^ and uACR of 50 mg/mmol	
PREDICT model: Probability of ≥ 25% reduction in eGFR or initiation of KRT between 6 wks and 12 mos postpartum			
26.3% (17.0%–38.4%)		45.9% (34.7%–57.8%)	
Kidney Failure Risk Equation (KFRE): Probability of requirement for KRT in 2 yrs			
2.09%		3.15%	

Patient C	Patient D	Patient E	Patient F
35-yr-old with preconception eGFR of 40 ml/min per 1.73 m^2^; Parity 2; No DM or transplant	35-yr-old with preconception eGFR of 40 ml/min per 1.73 m^2^; Parity 0; No DM with transplant	30-yr-old with preconception eGFR of 40 ml/min per 1.73 m^2^; Parity 2, with DM, no transplant	35-yr-old with preconception eGFR of 40 ml/min per 1.73m^2^; Parity 0; with DM, no transplant
PREDICT: Probability of SGA (< 3^rd^ percentile) infant and/or preterm delivery (< 34 wks’ gestation)			
34.7% (26.5%–43.8%)	52.4% (42.5%–62.0%)	54.5% (39.1%–69.0%)	58.1% (43.4%–71.3%)

DM, diabetes mellitus; eGFR, estimated glomerular filtration rate; KRT, kidney failure risk equation; SGA, small-for-gestational-age; uACR, urine albumin-to-creatinine ratio.

## Discussion

We have developed and validated 2 prediction models for estimating risk of pregnancy-associated progression of kidney disease and adverse neonatal outcomes in 3 international cohorts of women with CKD with replicated performance in sensitivity analyses; with outcomes codeveloped by women living with CKD of reproductive age, obstetricians, nephrologists, and midwives. The models have high sensitivity and NPV to provide reassurance to women at low risk of adverse outcomes and support clinicians to target antenatal care for higher risk women, but low specificity and PPV. To our knowledge, there is no accurate tool to predict kidney and neonatal outcomes to support individuals with CKD, as well as their partners and families to make complex life-changing decisions associated with pregnancy planning.

Historically, CKD was considered a contraindication to pregnancy; however, attitudes have evolved to provide shared reproductive choices, aligned with women’s priorities identified in questionnaire and qualitative studies.^16^^,^^17^ Current estimation of individual risk is derived from old, small cohorts which do not reflect current nephrology, obstetric, and neonatal practice. However, rates of pregnancy-associated progression of kidney disease in those with advanced disease in our cohorts are comparable with studies from several decades ago; however, live birth rates are substantially higher reflecting improved antenatal care.

Aligned with other risk prediction tools for assessing likelihood of future requirement for KRT outside of pregnancy,^18^^,^^19^ we report that younger age, proteinuria, and lower eGFR preconception were predictive of loss of ≥25% function, or initiation of KRT. Other studies have identified prepregnancy proteinuria to be an important predictor of pregnancy-associated progression of kidney disease.^5^^,^^20^, ^21^, ^22^ These findings support the use of therapies to reduce proteinuria (renin-angiotensin-aldosterone system blockade agents, sodium-glucose cotransporter 2 inhibitors), for CKD optimization before conception to modify risk of postpartum disease progression.^23^

We report for the first time that a functioning kidney transplant is protective for both the primary and secondary outcome, after adjustment of other risk factors. This may be attributable to those with kidney transplants being more likely to have prepregnancy counselling and optimization before pregnancy as well as increased frequency of monitoring compared with other pregnant women with CKD with similar prepregnancy eGFR. These findings are in keeping with a Dutch nationwide multicenter cohort study of 197 kidney transplant recipients which did not find a sustained deterioration in function postpartum.^24^ In contrast, in an Italian multicenter cohort study of 121 pregnancies in kidney transplant recipients and those with CKD stage 1, a greater proportion of kidney transplant recipients (*n* = 19) had a postpartum CKD stage shift compared with women without transplants (*n* = 610) (31.6% vs. 6.7%), but there were no differences at other CKD stages.^25^

The prevalence of chronic hypertension varied across the 3 cohorts (UK development: 60%; Ontario validation: 6%; UK validation: 55%). The lower prevalence in the Ontario validation cohort may reflect the higher baseline eGFR of this cohort and different coding practices for chronic diseases internationally. Despite multiple studies identifying preexisting chronic hypertension as a predictor of progression of CKD following pregnancy^5^ and outside of pregnancy,^26^ inclusion did not improve performance of either model. This may be attributable to the high prevalence of chronic hypertension in the development cohort; inadequate hypertensive control may have already been accounted for by proteinuria resulting in collinearity of the 2 variables, or use of hospital coding to confirm hypertension diagnosis may be incomplete.

In addition to lower age, lower eGFR preconception and absence of kidney transplant, higher parity and preexisting diabetes mellitus were predictive of preterm delivery before 34 weeks and/or a SGA infants. The inverse relationship between lower eGFR and risk of preterm delivery or SGA is well-recognized.^27^ Younger women are more likely to be affected by glomerulonephritides and associated inflammation may independently trigger placental insufficiency or spontaneous preterm delivery.^28^ In women without CKD, higher parity is associated with superior neonatal outcomes; however, a large Swedish population study reported that multiparous women with CKD had worse perinatal outcomes such as preterm birth and SGA infants, than nulliparous women with CKD after adjusting for age and BMI.^29^ The mechanisms may include repeated exacerbation of endothelial dysfunction during pregnancy leading to poor placental development in future pregnancies.

Women with diabetes are recognized to have substantially increased risk of adverse neonatal outcomes; and in keeping with our findings, those with diabetic kidney disease have recently been reported in a large metanalysis to have a nearly 7-fold greater risk of preterm delivery before 34 weeks and 10-fold greater risk of SGA neonates compared with those with nondiabetic CKD.^30^ Diabetes was not retained in the primary outcome model. This suggests that these outcomes may reflect different pathophysiology, and that the fetus may be more adversely affected by the diabetic milieu.

Potential to improve patient outcomes with individualized risk prediction is increasingly recognized^31^; and incorporation of risk prediction tools in both kidney and obstetric clinical practice guidelines are recommended to support decision making, standardize care, and enhance efficient resource allocation.^32^ Our codesigned pregnancy CKD models demonstrate the importance of pregnancy-specific risk estimation illustrated in Table 4. Although the models predict different outcomes (PREDICT: >25% loss of kidney function and progression to KRT within the first year postpartum; KFRE: KRT requirement within 2 years), for the group of women with complete data in the development cohort who required KRT within 2 years postpartum, there was a substantial difference between the PREDICT model and the KFRE risk estimations (48% v 1.4%). In addition, there was a considerable disparity between risk estimates for the individual (e.g., 45.9% vs. 3.1%) (Table 4). This comparison is limited by the small sample size and difference in outcome but does suggest caution using KFRE to predict overall risk in those who have had a pregnancy.

Our findings also highlight that many women with eGFR < 90 ml/min per 1.73 m^2^ can be reassured that they are likely to have a favorable outcome: 70% of the development cohort and 97.1% and 80.5% of the validation cohorts did not have a 25% decline in eGFR or require KRT; 74% of the development cohort and 92.9% and 76.1% of the validation cohorts delivered after 34 weeks and did not have a SGA infant. Use of these prediction tools may improve service delivery efficiency, because some women with CKD can be deescalated to less resource-intensive pathways. The use of this model will mean a large proportion of women with normal outcomes will be considered high risk due to low PPVs in the model; however, surveillance for development of complications, including preeclampsia, fetal growth restriction, disease flare, and progression are still needed to ensure optimal outcomes.

Pregnancy may be the first time a woman interacts with health care and thus offers an opportunity to address health care inequalities. Women from ethnic minorities and those from socioeconomic deprivation are more likely to develop CKD and to have pregnancies with advanced CKD.^33^ In addition they are most likely to be negatively affected by restrictions of reproductive rights and least likely to receive prepregnancy counselling.^34^ Risk prediction would allow nephrologists to provide greater advocacy, enhanced support, and/or reassurance for these high-risk groups.

Incomplete data, including insufficient etiologies of kidney disease, laboratory data, and comorbidities may have impacted on findings; however, the use of routinely collected clinical and laboratory data will enhance the ability to implement. There was no evidence of an association between BMI and the primary or secondary outcome among the women with data. With such poor completeness, it was plausible that the presence of BMI data could be associated with the BMI value itself, even conditional on the complete data, thus violating the Missing at Random assumption required for multiple imputation. We therefore excluded this as a candidate variable, but acknowledge that having complete and accurate BMI data may have resulted in an improved predictive performance. Another limitation of this study is the inclusion of cohorts from high-income countries with access to universal health care, prohibiting generalizability to other settings. Low specificity is likely to lead to heightened antenatal surveillance inappropriately for women with higher risk because many will still have successful pregnancies, and this is reflected by the low event rate in the validation cohorts. However, the strength of this tool is that it will enable reassurance of many women who will have successful pregnancy outcomes who may previously have been discouraged from conceiving and achieving their pregnancy wish.

In conclusion, we have codeveloped and validated accurate predictive models for loss of kidney function postpartum, preterm delivery, and SGA infants using readily available data. These tools have the potential to provide women with CKD with individualized risk of adverse events to empower women to make informed choices about pregnancy and support health care professionals to give accurate prepregnancy counselling and stratified obstetric care. We also highlight potential limitations of KFRE in women of reproductive age, if pregnancy events are included in risk prediction. Impact evaluation, including for marginalized groups and further optimization external validation in low resource settings with greatest burden of risk is now needed.

## Appendix

### List of the PREDICT Investigation Group

Elizabeth Ralston, Kings College London; Kate Wiles, Bart’s and the London NHS Foundation Trust; Michelle Hladunewich, University of Toronto; Yanzhong Wang, Kings College London; Amanda Clery, University College London; Joseph Chilcot, Kings College London; Chris Farmer, University of Kent; Steve Childs, University of Kent, Juan-Jesus Carrero, Karolinska Institutet; Yuanhang Yang, Karolinska Institutet; Nivethika Jeyakumar, ICES; Amit Garg, ICES; Lavanya Bathini, University of Alberta; Graham Smith, ICES; Hannah Blakey, Queen Elizabeth Hospital Birmingham; Nadia Sarween, Queen Elizabeth Hospital Birmingham; Graham Lipkin, Queen Elizabeth Hospital Birmingham; Ellen Knox, Birmingham Women’s and Children’s NHS Foundation Trust; Tess Harris, Polycystic Kidney Disease Charity; David Pitcher, The UK Kidney Association; Shalini Santhakumaran, The UK Kidney Association; Anna Casula, The UK Kidney Association; Retha Steenkamp, The UK Kidney Association; Lucy Chappell, King’s College London; Philip Webster, Imperial College Healthcare NHS Trust; Sue Carr, University Hospitals of Leicester NHS Trust; Matthew Hall, Nottingham University Hospitals; Liz Lightstone, Imperial College London; and Kate Bramham: Kings College London.

## Disclosure

All the authors declared no competing interests.

## Supplementary Material

Supplementary File (PDF) **Table S1.** Missing data within the overall PREDICT cohort. **Table S2.** Prediction model parameters from multivariable regression. **Table S3.** Model performance measures for primary and secondary outcomes. **Table S4.** Characteristics of women stratified by the primary and secondary outcome. TRIPOD checklist.

## Footnotes

Supplementary File (PDF)
**Table S1.** Missing data within the overall PREDICT cohort. **Table S2.** Prediction model parameters from multivariable regression. **Table S3.** Model performance measures for primary and secondary outcomes. **Table S4.** Characteristics of women stratified by the primary and secondary outcome. TRIPOD checklist.

## References

[1] Mills K.T., Xu Y., Zhang W. (2015). A systematic analysis of worldwide population-based data on the global burden of chronic kidney disease in 2010. Kidney Int.

[2] Wiles K., Chappell L., Clark K. (2019). Clinical practice guideline on pregnancy and renal disease. BMC Nephrol.

[3] Centers for Disease Control and Prevention (2023). Chronic kidney disease in the United States. US Department of Health and Human Services, Centers for Disease Control and Prevention. https://www.cdc.gov/kidney-disease/php/data-research/index.html.

[4] Cabiddu G., Castellino S., Gernone G. (2016). A best practice position statement on pregnancy in chronic kidney disease: the Italian Study Group on Kidney and Pregnancy. J Nephrol.

[5] Wiles K., Webster P., Seed P.T. (2021). The impact of chronic kidney disease Stages 3–5 on pregnancy outcomes. Nephrol Dial Transplant.

[6] de Jong M.F.C., van Hamersvelt H.W., van Empel I.W.H., Nijkamp E.J.W., Lely A.T., Dutch Guideline Working Group on Pregnancy in CKD (2022). Summary of the Dutch practice guideline on pregnancy wish and pregnancy in CKD. Kidney Int Rep.

[7] Bramham K., Seed P.T., Lightstone L. (2016). Diagnostic and predictive biomarkers for pre-eclampsia in patients with established hypertension and chronic kidney disease. Kidney Int.

[8] Sarween N., Hodson J., Knox E., Day C., Drayson M., Lipkin G. (2019). A study of maternal and fetal outcomes in a cohort of pregnant women with chronic kidney disease. Nephrol Dial Transplant.

[9] Heaman M., Gupton A. (2009). Psychometric testing of the perception of pregnancy risk questionnaire. Res Nurs Health.

[10] Harel Z., McArthur E., Hladunewich M. (2019). Serum creatinine levels before, during, and after pregnancy. JAMA.

[11] Levey A., Stevens L., Schmid C. (2009). A new equation to estimate glomerular filtration rate. Ann Intern Med.

[12] Villar J., Cheikh Ismail L., Victora C.G. (2014). International standards for newborn weight, length, and head circumference by gestational age and sex: the Newborn Cross-Sectional Study of the INTERGROWTH-21st Project. Lancet.

[13] Ensor J., Martin E., Riley R. (2021). Pmsampsize: calculates the minimum sample size required for developing a multivariable prediction model. https://cran.r-hub.io/web/packages/pmsampsize/pmsampsize.pdf.

[14] Moons K.G.M., Altman D.G., Reitsma J.B. (2015). Transparent reporting of a multivariable prediction model for individual prognosis or diagnosis (TRIPOD): explanation and elaboration. Ann Intern Med.

[15] Wood A.M., White I.R., Royston P. (2008). How should variable selection be performed with multiply imputed data?. Stat Med.

[16] Tong A., Brown M.A., Winkelmayer W.C., Craig J.C., Jesudason S. (2015). Perspectives on pregnancy in women with CKD: a semistructured interview study. Am J Kidney Dis.

[17] Ralston E., Bramham K. (2022). MO1044: understanding perception of pregnancy risk in women with chronic kidney disease. Nephrol Dial Transplant.

[18] Johnson E.S., Thorp M.L., Platt R.W., Smith D.H. (2008). Predicting the risk of dialysis and transplant among patients with CKD: a retrospective cohort study. Am J Kidney Dis.

[19] Tangri N., Stevens L.A., Griffith J., Tighiouart H., Djurdjev O., Naimark D. (2011). A predictive model for progression of chronic kidney disease to kidney failure. JAMA.

[20] Imbasciati E., Gregorini G., Cabiddu G. (2007). Pregnancy in CKD stages 3 to 5: fetal and maternal outcomes. Am J Kidney Dis.

[21] Oh H.J., Han S.H., Yoo D.E. (2011). Reduced pre-pregnancy proteinuria is associated with improving postnatal maternal renal outcomes in IgA nephropathy women. Clin Nephrol.

[22] Tangren J., Bathini L., Jeyakumar N. (2023). Pre-pregnancy eGFR and the risk of adverse maternal and fetal outcomes: a population-based study. J Am Soc Nephrol.

[23] Sarafidis P., Ortiz A., Ferro C.J. (2021). ‘Hypertension and the Kidney’ working group of the European Society of Hypertension (ESH) and the ‘European Renal and cardiovascular Medicine’ (EURECA-m) working group of the European Renal Association–European Dialysis and Transplant Association (ERA-EDTA). Sodium--glucose co-transporter-2 inhibitors for patients with diabetic and nondiabetic chronic kidney disease: a new era has already begun. J Hypertens.

[24] van Buren M.C., Gosselink M., Groen H. (2022). Effect of pregnancy on eGFR after kidney transplantation: a national cohort study. Transplantation.

[25] Piccoli G.B., Cabiddu G., Attini R. (2017). Outcomes of pregnancies after kidney transplantation: lessons learned from CKD. A comparison of transplanted, nontransplanted chronic kidney disease patients and low-risk pregnancies: a multicenter nationwide analysis. Transplantation.

[26] Yu Z., Rebholz C.M., Wong E. (2019). Association between hypertension and kidney function decline: the atherosclerosis risk in communities (ARIC) study. Am J Kidney Dis.

[27] Smith P.A., Sarris I., Clark K., Wiles K., Bramham K. (2025). Kidney disease and reproductive health. Nat Rev Nephrol.

[28] Hui D., Hladunewich M.A. (2019). Chronic kidney disease and pregnancy. Obstet Gynecol.

[29] Khalaf S.Y., O’Reilly É.J., McCarthy F.P., Kublickas M., Kublickiene K., Khashan A.S. (2021). Pregnancy outcomes in women with chronic kidney disease and chronic hypertension: a National cohort study. Am J Obstet Gynecol.

[30] Jeyaraman D., Walters B., Bramham K., Fish R., Lambie M., Wu P. (2024). Adverse pregnancy outcomes in pregnant women with chronic kidney disease: A systematic review and meta-analysis. BJOG.

[31] Tripepi G., Heinze G., Jager K.J., Stel V.S., Dekker F.W., Zoccali C. (2013). Risk prediction models. Nephrol Dial Transplant.

[32] Kidney Disease: Improving Global Outcomes (KDIGO) CKD Work Group (2024). KDIGO 2024 clinical practice guideline for the evaluation and management of chronic kidney disease. Kidney Int.

[33] Shah S., Christianson A.L., Meganathan K., Leonard A.C., Schauer D.P., Thakar C.V. (2019). Racial differences and factors associated with pregnancy in ESKD patients on dialysis in the United States. J Am Soc Nephrol.

[34] Rizzolo K., Faucett A., Kendrick J. (2023). Implications of antiabortion laws on patients with kidney disease in pregnancy. Clin J Am Soc Nephrol.

