# Supplementary material for: PREDICT Tool for Pregnancy-Associated CKD Progression
Source: Kidney Int Rep. 2026 Jun 6;11(9):106636. doi: 10.1016/j.ekir.2026.106636 (PMC13356684; doi:10.1016/j.ekir.2026.106636)

**Supplementary Appendix**

**Table of Contents**

Acknowledgements……………………………………………………………………………2

Table S1: Missing data within the overall PREDICT cohort…………………………………10

Table S2: Prediction model parameters from multivariable regression……………………….11

Table S3: Model performance measures for primary and secondary outcomes………………12

Table S4: Characteristics of women stratified by the primary and secondary outcome…….....17

TRIPOD Checklist……………………………………………………………………………20

**Acknowledgements**

PREDICT Investigation Group: Elizabeth Ralston: Kings College London, Kate Wiles: Bart’s and the London NHS Foundation Trust, Michelle Hladunewich: University of Toronto, Yanzhong Wang: Kings College London, Amanda Clery: University College London, Joseph Chilcot: Kings College London, Chris Farmer: University of Kent, Steve Childs: University of Kent, Juan-Jesus Carrero: Karolinska Institutet, Yuanhang Yang: Karolinska Institutet, Nivethika Jeyakumar: ICES, Amit Garg: ICES, Lavanya Bathini: University of Alberta, Graham Smith: ICES, Hannah Blakey: Queen Elizabeth Hospital Birmingham, Nadia Sarween: Queen Elizabeth Hospital Birmingham, Graham Lipkin: Queen Elizabeth Hospital Birmingham, Ellen Knox: Birmingham Women’s and Children’s NHS Foundation Trust, Tess Harris: Polycystic Kidney Disease Charity, David Pitcher: The UK Kidney Association, Shalini Santhakumaran: The UK Kidney Association, Anna Casula: The UK Kidney Association, Retha Steenkamp: The UK Kidney Association, Lucy Chappell: King’s College London, Philip Webster: Imperial College Healthcare NHS Trust, Sue Carr: University Hospitals of Leicester NHS Trust, Matthew Hall: Nottingham University Hospitals, Liz Lightstone: Imperial College London, Kate Bramham: Kings College London.

RaDaR Consortium: Sharirose Abat: St George’s University Hospitals NHS Foundation Trust, UK, Shazia Adalat: Evelina London Children's Hospital, UK, Joy Agbonmwandolor: David Evans Medical Research Centre, Nottingham University Hospitalss NHS Trust, UK, Zubaidah Ahmad: Guy's and St Thomas NHS Foundation Trust, UK, Abdulfattah Alejmi: Ysbyty Gwynedd, Betsi Cadwaladr University Health Board, UK, Rashid Almasarwah: Imperial College Healthcare NHS Trust, UK, Nicholas Annear: St George’s University Hospitals NHS Foundation Trust, UK, Zainab Arslan: UCL Great Ormond Street Institute of Child Health, UK, Ellie Asgari: Guy's and St Thomas NHS Foundation Trust, UK, Amanda Ayers: James Paget University Hospital NHS Foundation Trust, UK, Jyoti Baharani: Heart of England NHS Foundation Trust, Birmingham, UK, Gowrie Balasubramaniam: Mid and South Essex NHS Foundation Trust, UK, Tarun Bansal: Bradford Teaching Hospitals NHS Foundation Trust, UK, Alison Barratt: Royal United Hospital Bath NHS Trust, UK, Jonathan Barratt: University of Leicester, UK, Megan Bates: Freeman Hospital, Newcastle Upon Tyne, UK, Natalie Bayne: Birmingham Women's and Children's NHS Foundation Trust, UK, Janet Bendle: Manchester University NHS Foundation Trust, UK, Sarah Benyon: Royal Devon University Healthcare NHS Foundation Trust, UK, Carsten Bergmann: Medizinische Genetik Mainz, Mainz, Germany, Sunil Bhandari: Hull University Teaching Hospitals NHS Trust, UK, Coralie Bingham: Exeter Kidney Unit, Royal Devon University Healthcare NHS Foundation Trust, UK, Preetham Boddana: Gloucestershire Hospitals NHS Foundation Trust, UK, Sally Bond: Oxford University Hospitals NHS Foundation Trust, UK, Fiona Braddon: UK Kidney Association, UK, Kate Bramham: UK Kidney Association, UK, Angela Branson: Manchester University NHS Foundation Trust, UK, Stephen Brearey: Countess of Chester NHS Foundation Trust, UK, Vicky Brocklebank: National Renal Complement Therapeutics Centre, Newcastle upon Tyne Hospitals NHS Foundation Trust, Newcastle upon Tyne, UK, Sharanjit Budwal: University Hospitals of Leicester NHS Trust, UK, Conor Byrne: Barts Health NHS Trust, London, UK, Hugh Cairns: King's College Hospital NHS Foundation Trust, UK, Brian Camilleri: East Suffolk and North Essex NHS Foundation Trust, UK, Gary Campbell: Ninewells Hospital and Medical School, Dundee, UK, Alys Capell: North West Anglia NHS Foundation Trust, UK, Margaret Carmody: Heart of England NHS Foundation Trust, Birmingham, UK, Marion Carson: Northern Health and Social Care Trust and Northern Ireland Clinical Research Network, Tracy Cathcart: Hull University Teaching Hospitals NHS Trust, UK, Christine Catley: Mid and South Essex NHS Foundation Trust, UK, Karine Cesar: West Suffolk NHS Foundation Trust, UK, Melanie Chan: Imperial College Healthcare NHS Trust, UK, Houda Chea: Manchester University NHS Foundation Trust, UK, James Chess: Morriston Hospital, Swansea Bay Health Board, UK, Chee Kay Cheung: University Hospitals of Leicester NHS Trust, UK, Katy-Jane Chick: Lister Hospital, East and North Hertfordshire NHS Trust, UK, Nihil Chitalia: Dartford and Gravesham NHS Trust, UK, Martin Christian: Nottingham Children’s Hospital, UK, Tina Chrysochou: Salford Royal Hospital, Northern Care Alliance NHS Foundation Trust, Salford, UK, Katherine Clark: King’s College London, UK, Christopher Clayton: Nottingham University Hospitalss NHS Trust, UK, Rhian Clissold: Exeter Kidney Unit, Royal Devon University Healthcare NHS Foundation Trust, UK, Helen Cockerill: West Suffolk NHS Foundation Trust, UK, Joshua Coelho: Epsom and St Helier University Hospitals NHS Trust, UK, Elizabeth Colby: University of Bristol Medical School, Bristol, UK, Viv Colclough: Royal Stoke University Hospital, UK, Eileen Conway: Manchester Royal Infirmary, UK, H. Terence Cook: Centre for Inflammatory Disease, Imperial College London, UK, Wendy Cook: Nephrotic Syndrome Trust (NeST), UK, Theresa Cooper: North Cumbria Integrated Care NHS Foundation Trust, UK, Richard J Coward: University of Bristol Medical School, Bristol, UK, Sarah Crosbie: Oxford University Hospitals NHS Foundation Trust, UK, Gabor Cserep: Colchester General Hospital, UK, Sunil Daga: Leeds Teaching Hospitals NHS Trust, UK, Anjali Date: Tameside and Glossop Integrated Care NHS Foundation Trust, UK, Katherine Davidson: North Cumbria Integrated Care NHS Foundation Trust, UK, Amanda Davies: Wye Valley NHS Trust, UK, Neeraj Dhaun: BHF Centre for Cardiovascular Science, The Queen's Medical Research Institute, University of Edinburgh, UK, Ajay Dhaygude: Lancashire Teaching Hospital, UK, Lynn Diskin: Royal United Hospital Bath NHS Trust, UK, Abhijit Dixit: Nottingham University Hospitalss NHS Trust, UK, Eunice Ann Doctolero: Lister Hospital, East and North Hertfordshire NHS Trust, UK, Suzannah Dorey: Leeds Teaching Hospitals NHS Trust, UK, Lewis Downard: UK Kidney Association, UK, Mark Drayson: University of Birmingham, UK, Gavin Dreyer: Barts Health NHS Trust, London, UK, Tina Dutt: Liverpool University Hospitals Foundation NHS Trust, UK, Kufreabasi Etuk: King's College Hospital NHS Foundation Trust, UK, Dawn Evans: Salford Royal NHS Foundation Trust, UK, Jenny Finch: East Suffolk and North Essex NHS Foundation Trust, UK, Frances Flinter: Department of Clinical Genetics, Guy's and St Thomas’ NHS Foundation Trust, UK, James Fotheringham: Centre for Health and Related Research, School of Population Health, University of Sheffield, UK, Lucy Francis: Department of Medicine, University of Cambridge, UK, Fiona Karet Frankl: Department of Medicine, University of Cambridge, UK, Daniel P. Gale: University College London Department of Renal Medicine, Royal Free Hospital, UK, Hugh Gallagher: SW Thames Renal Unit, Epsom and St Helier University Hospitals NHS Trust, UK, David Game: Guy's and St Thomas NHS Foundation Trust, UK, Eva Lozano Garcia: Epsom and St Helier University Hospitals NHS Trust, UK, Madita Gavrila: Oxford University Hospitals NHS Foundation Trust, UK, Susie Gear: Alport UK, UK, Colin Geddes: Queen Elizabeth University Hospital, Glasgow, UK, Mark Gilchrist: College of Medicine and Health, University of Exeter, UK, Matt Gittus: Division of Population Health, University of Sheffield, UK, Paraskevi Goggolidou: University of Wolverhampton, UK, Christopher Goldsmith: Liverpool University Hospitals Foundation NHS Trust, UK, Patricia Gooden: Patient Representative, UK, Andrea Goodlife: University Hospitals of Leicester NHS Trust, UK, Priyanka Goodwin: Lancashire Teaching Hospital, UK, Tassos Grammatikopoulos: King's College Hospital NHS Foundation Trust, UK, Barry Gray: Sheffield Kidney Institute, Sheffield Teaching Hospitals NHS Foundation Trust, UK, Megan Griffith: Centre for Inflammatory Disease, Imperial College London, UK, Steph Gumus: Mid and South Essex NHS Foundation Trust, UK, Sanjana Gupta: Royal Free Hospital, UK, Patrick Hamilton: Manchester Institute of Nephrology and Transplantation, Manchester Royal Infirmary, UK, Lorraine Harper: University of Birmingham, UK, Tess Harris: PKD Charity, UK, Louise Haskell: University Hospitals Southampton NHS Foundation Trust, UK, Samantha Hayward: University of Bristol Medical School, Bristol, UK, Shivaram Hegde: Children's Kidney Centre, University Hospital of Wales, UK, Bruce Hendry: Travere Therapeutics, UK, Sue Hewins: University Hospitals Coventry and Warwickshire NHS Trust, UK, Nicola Hewitson: County Durham & Darlington NHS Foundation Trust, UK, Kate Hillman: Manchester University NHS Foundation Trust, UK, Mrityunjay Hiremath: Liverpool University Hospitals Foundation NHS Trust, UK, Alexandra Howson: University of Leicester, UK, Zay Htet: King's College Hospital NHS Foundation Trust, UK, Sharon Huish: Royal Devon University Healthcare NHS Foundation Trust, UK, Richard Hull: St George’s University Hospitals NHS Foundation Trust, UK, Alister Humphries: Patient Representative, UK, David P. J. Hunt: University of Edinburgh, UK, Karl Hunter: Wirral University Teaching Hospital NHS Foundation Trust, UK, Samantha Hunter: Hull University Teaching Hospitals NHS Trust, UK, Marilyn Ijeomah-Orji: Imperial College Healthcare NHS Trust, UK, Nick Inston: University Hospitals Birmingham NHS Foundation Trust, UK, Chadd Javier: East and North Hertfordshire NHS Trust, UK, David Jayne: Department of Medicine, University of Cambridge, UK, Gbemisola Jenfa: North West Anglia NHS Foundation Trust, UK, Alison Jenkins: North Bristol NHS Trust, UK, Sally Johnson: Great North Children's Hospital, Newcastle Upon Tyne, UK, Amanda Jones: Ysbyty Gwynedd, Betsi Cadwaladr University Health Board, UK, Caroline A Jones: Alder Hey Children's NHS Foundation Trust, UK, Colin Jones: York & Scarborough Teaching Hospitals NHS Foundation Trust, UK, Rachel Jones: Department of Medicine, University of Cambridge, UK, Lavanya Kamesh: University Hospitals Birmingham NHS Foundation Trust, UK, Durga Kanigicherla: University of Manchester, UK, Mahzuz Karim: Norfolk and Norwich University Hospitals NHS Trust, UK, Amrit Kaur: Royal Manchester Children’s Hospital, Manchester, UK, David Kavanagh: National Renal Complement Therapeutics Centre, Newcastle upon Tyne Hospitals NHS Foundation Trust, Newcastle upon Tyne, UK, Kelly Kearley: PTEN UK and Ireland Patient Group, Larissa Kerecuk: Birmingham Women's and Children's NHS Foundation Trust, UK, Arif Khwaja: Sheffield Kidney Institute, Sheffield Teaching Hospitals NHS Foundation Trust, UK, Garry King: UK Kidney Association, UK, Ewa Kislowska: Guy's and St Thomas NHS Foundation Trust, UK, Edyta Klata: East Suffolk and North Essex NHS Foundation Trust, UK, Maria Kokocinska: Birmingham Women's and Children's NHS Foundation Trust, UK, Felix Jo-Bamba Kpodo: Royal Berkshire NHS Foundation Trust, UK, Ling Wai Maggie Lai: Royal Devon University Healthcare NHS Foundation Trust, UK, Ania Koziell Mark Lambie: School of Medicine, Keele University, UK, Laura Lawless: Nottingham University Hospitalss NHS Trust, UK, Thomas Ledson: Wirral University Teaching Hospital NHS Foundation Trust, UK, Rachel Lennon: Wellcome Centre for Cell-Matrix Research, University of Manchester, UK, Adam P Levine: Research Department of Pathology, University College London, UK, Andrew Lewington: Leeds Teaching Hospitals NHS Trust, UK, Graham Lipkin: University Hospitals Birmingham NHS Foundation Trust, UK, Graham Lovitt: HLRCC Foundation, UK, Paul Lyons: Cambridge Institute of Therapeutic Immunology and Infectious Disease, Cambridge, UK, Holly Mabillard: Newcastle University, UK, Katherine Mackintosh: James Paget University Hospital NHS Foundation Trust, UK, Khalid Mahdi: United Lincolnshire Hospitals NHS Trust, UK, Eamonn Maher: Department of Medical Genetics, University of Cambridge, UK, Kevin J. Marchbank: National Renal Complement Therapeutics Centre, Newcastle upon Tyne Hospitals NHS Foundation Trust, Newcastle upon Tyne, UK, Patrick B Mark: Queen Elizabeth University Hospital, Glasgow, UK, Sherry Masoud: UK Kidney Association, UK, Bridgett Masunda: Mid and South Essex NHS Foundation Trust, UK, Zainab Mavani: North West Anglia NHS Foundation Trust, UK, Jake Mayfair: Guy's and St Thomas NHS Foundation Trust, UK, Stephen McAdoo: Imperial College Healthcare NHS Trust, UK, Joanna Mckinnell: University Hospitals of Derby and Burton NHS Foundation Trust, UK, Nabil Melhem: Evelina London Children's Hospital, UK, Simon Meyrick: Wye Valley NHS Trust, UK, Shabbir Moochhala: University College London Department of Renal Medicine, Royal Free Hospital, UK, Ann Morgan: National Institute of Health and Care Research Leeds Biomedical Research Centre, Leeds Teaching Hospitals NHS Trust, UK, Fawad Muhammad: Ysbyty Gwynedd, Betsi Cadwaladr University Health Board, UK, Shona Murray: Ninewells Hospital and Medical School, Dundee, UK, Kristina Novobritskaya: Oxford University Hospitals NHS Foundation Trust, UK, Albert CM Ong: Division of Population Health, University of Sheffield, UK, Louise Oni: University of Liverpool, UK, Kate Osmaston: UK Kidney Association, UK, Neal Padmanabhan: Queen Elizabeth University Hospital, Glasgow, UK, Sharon Parkes: Birmingham Women's and Children's NHS Foundation Trust, UK, Jean Patrick: James Paget University Hospital NHS Foundation Trust, UK, James Pattison: Guy's and St Thomas NHS Foundation Trust, UK, Riny Paul: St George’s University Hospitals NHS Foundation Trust, UK, Rachel Percival: Newcastle Upon Tyne Hospitals NHS Foundation Trust, UK, Stephen J. Perkins: Research Department of Structural and Molecular Biology, University College London, UK, Alexandre Persu: Division of Cardiology, Cliniques Universitaires Saint-Luc, Belgium, William G Petchey: Cambridge University Hospitals NHS Foundation Trust, UK, Matthew C. Pickering: Centre for Inflammatory Disease, Imperial College London, UK, Jennifer Pinney: University Hospitals Birmingham NHS Foundation Trust, UK, David Pitcher: UK Kidney Association, UK, Lucy Plumb: University of Bristol Medical School, Bristol, UK, Zoe Plummer: UK Kidney Association, UK, Joyce Popoola: St George’s University Hospitals NHS Foundation Trust, UK, Frank Post: King's College Hospital NHS Foundation Trust, UK, Albert Power: North Bristol NHS Trust, UK, Guy Pratt: University of Birmingham, UK, Charles Pusey: Centre for Inflammatory Disease, Imperial College London, UK, Morgan Putnam: Retroperitoneal Fibrosis (RF) Group, UK, Susan Pywell: UK Kidney Association, UK, Ria Rabara: Oxford University Hospitals NHS Foundation Trust, UK, May Rabuya: Guy's and St Thomas NHS Foundation Trust, UK, Tina Raju: Epsom and St Helier University Hospitals NHS Trust, UK, Ian SD Roberts: Oxford University Hospitals NHS Foundation Trust, UK, Candice Roufosse: Department of Immunology and Inflammation, Faculty of Medicine, Imperial College London, UK, Adam Rumjon: King's College Hospital NHS Foundation Trust, UK, Alan Salama: University College London Department of Renal Medicine, Royal Free Hospital, UK, Moin Saleem: University of Bristol Medical School, Bristol, UK, RN Sandford: Department of Medical Genetics, University of Cambridge, UK, Kanwaljit S. Sandu: Shrewsbury and Telford Hospital NHS Trust, UK, Nadia Sarween: University Hospitals Birmingham NHS Foundation Trust, UK, John A. Sayer: Newcastle University, UK, Neil Sebire: National Institute of Health and Care Research Great Ormond Street Hospital Biomedical Research Centre, UK, Haresh Selvaskandan: University Hospitals of Leicester NHS Trust, UK, Sapna Shah: King's College Hospital NHS Foundation Trust, UK, Asheesh Sharma: Liverpool University Hospitals Foundation NHS Trust, UK, Edward J Sharples: Oxford University Hospitals NHS Foundation Trust, UK, Neil Sheerin: National Renal Complement Therapeutics Centre, Newcastle upon Tyne Hospitals NHS Foundation Trust, Newcastle upon Tyne, UK, Harish Shetty: Lancashire Teaching Hospital, UK, Rukshana Shroff: UCL Great Ormond Street Institute of Child Health, UK, Roslyn Simms: Sheffield Kidney Institute, Sheffield Teaching Hospitals NHS Foundation Trust, UK, Manish Sinha: Evelina London Children's Hospital, UK, Smeeta Sinha: Northern Care Alliance NHS Foundation Trust, UK, Kerry Smith: East Suffolk and North Essex NHS Foundation Trust, UK, Lara Smith: Manchester University NHS Foundation Trust, UK, Shalabh Srivastava: South Tyneside and Sunderland NHS Foundation Trust, UK, Retha Steenkamp: UK Kidney Association, UK, Ian Stott: Doncaster and Bassetlaw Teaching Hospitals, UK, Katerina Stroud: Department of Medical Genetics, University of Cambridge, UK, Pauline Swift: Epsom and St Helier University Hospitals NHS Trust, UK, Justyna Szklarzewicz: University Hospitals of Leicester NHS Trust, UK, Fred Tam: Centre for Inflammatory Disease, Imperial College London, UK, Kay Tan: New Cross Hospital, Wolverhampton, UK, Robert Taylor: Wellcome Centre for Mitochondrial Research, Translational & Clinical Research Institute, Faculty of Medical Sciences, Newcastle University, UK, Kay Thomas: Guy's and St Thomas NHS Foundation Trust, UK, Marc Tischkowitz: Department of Medical Genetics, University of Cambridge, UK, Yincent Tse: Great North Children's Hospital, Newcastle Upon Tyne, UK, Alison Turnbull: York & Scarborough Teaching Hospitals NHS Foundation Trust, UK, A. Neil Turner: University of Edinburgh, UK, Kay Tyerman: Leeds Teaching Hospitals NHS Trust, UK, Miranda Usher: Calderdale & Huddersfield Foundation Trust, UK, Gopalakrishnan Venkat-Raman: Royal Surrey County Hospital, Guildford, UK, Alycon Walker: South Tees Hospitals NHS Foundation Trust, UK, Stephen B. Walsh: University College London Department of Renal Medicine, Royal Free Hospital, UK, Aoife Waters: University College Cork, Ireland, Angela Watt: Patient Representative, UK, Phil Webster: Imperial College Healthcare NHS Trust, UK, Ashutosh Wechalekar: National Amyloidosis Centre, University College London, UK, Gavin Iain Welsh: University of Bristol Medical School, Bristol, UK, Nicol West: Great Western Hospital, Swindon, UK, David Wheeler: University College London Department of Renal Medicine, Royal Free Hospital, UK, Kate Wiles: Barts Health NHS Trust, London, UK, Lisa Willcocks: Cambridge University Hospitals NHS Foundation Trust, UK, Angharad Williams: West Suffolk NHS Foundation Trust, UK, Emma Williams: East Suffolk and North Essex NHS Foundation Trust, UK, Karen Williams: Guy's and St Thomas NHS Foundation Trust, UK, Deborah H Wilson: North Tees and Hartlepool NHS Foundation Trust, UK, Patricia D. Wilson: University College London, UK, Paul Winyard: Mid and South Essex NHS Foundation Trust, UK, Edwin Wong: National Renal Complement Therapeutics Centre, Newcastle upon Tyne Hospitals NHS Foundation Trust, Newcastle upon Tyne, UK, Katie Wong: UK Kidney Association, UK, Grahame Wood: Salford Royal NHS Foundation Trust, UK, Emma Woodward: Manchester University NHS Foundation Trust, UK, Len Woodward: aHUS Alliance, UK, Adrian Woolf: School of Biological Sciences, University of Manchester, UK, David Wright: Royal Free Hospital, UK

**Table S1: Missing data within the overall PREDICT cohort**

The variables that have missingness:

| Missing (N) (%) | Development Cohort  n= 746 | Validation Cohort- Ontario, Canada  n= 6974 | Validation Cohort- UK  n= 380 |
| --- | --- | --- | --- |
| Ethnicity | 4 (0.5%) | 6974 (100%) | 2 (0.5%) |
| Parity | 8 (1.1) | 0 (0%) | 23 (6.1%) |
| Maternal BMI | 571 (77%) | 6974 (100%) | 48 (12.6%) |
| CKD cause | 25 (3.4%) | 6,197 (88.9%) | 7 (1.8%) |
| Pre-pregnancy diabetes | 0 (0%) | 0 (0%) | 255 (67.1%) |
| Postpartum eGFR or KRT | 0 (0%) | 0 (0%) | 120 (31.6%) |
| Gestation weeks | 168 (22.5%) | 0 (0%) | 8 (2.1%) |
| Proteinuria | 376 (50.4) | 0 (0%) | 137 (36.1%) |

**Table S2: Prediction model parameters from multivariable regression**

|  |  | **Multiple imputation** | | **Complete case** | |
| --- | --- | --- | --- | --- | --- |
| **Primary outcome** |  | coefficient | SE | coefficient | SE |
| Intercept |  | 1.9119 | 0.6225 | 3.4051 | 1.0119 |
| Age at conception | Per 1 year increase in age | -0.0471 | 0.0174 | -0.0846 | 0.0274 |
| Proteinuria | Moderate | -0.1377 | 0.1612 | -0.2392 | 0.1964 |
|  | Severe | 0.5316 | 0.1803 | 0.6948 | 0.1753 |
| Transplant |  | -0.2004 | 0.0921 | - | - |
| eGFR pre-conception (ml/min/1.73 m2) | Per 1 unit increase in eGFR | -0.0239 | 0.0049 | -0.0325 | 0.00769 |
|  |  |  |  |  |  |
| **Secondary outcome** |  |  |  |  |  |
| Intercept |  |  |  | 2.4067 | 0.76 |
| Age at conception | Per 1 year increase in age |  |  | -0.0431 | 0.0208 |
| Transplant |  |  |  | 0.3711 | 0.1043 |
| eGFR pre-conception (ml/min/1.73 m2) | Per 1 unit increase in eGFR |  |  | -0.0293 | 0.00589 |
| Multiparous |  |  |  | -0.3602 | 0.1034 |
| Diabetes |  |  |  | 0.599 | 0.1576 |

e.g. the predicted probability of the primary outcome (reduction in eGFR of 25% or more, or starting KRT, within 1 year of delivery) from the multiple imputation model for a transplant patient age 35 with moderate proteinuria and a pre-conception eGFR of 40:

probability = exp(xb) / (1+exp(xb))

xb = 1.9119 + 35*-0.0471 - 0.1377 - 0.2004 + 40*-0.0239

= -1.0307

probability = exp(-1.0307) / (1+exp(-1.0307)) = 0.263

**Table S3a: Model performance measures for primary and secondary outcomes for development cohort**

|  | | **Development cohort** | | | | | |
| --- | --- | --- | --- | --- | --- | --- | --- |
| **Primary outcome** | | **N** | **AUROC (95% CI)** | **R^2^** | **Brier score** | **Calibration intercept** | **Calibration slope** |
| **Multiple imputation** | **All** | 746 | 0.70 (0.66, 0.74) | 0.15 | 0.19 | 0 (-0.244, 0.248) | 0.996 (0.771, 1.23) |
|  | **eGFR<60** | 405 | 0.71 (0.66, 0.77) |  |  | 0.062 (-0.208, 0.337) | 1.149 (0.826, 1.49) |
|  | **eGFR<45** | 196 | 0.73 (0.66, 0.8) |  |  | 0 (-0.317, 0.323) | 1.373 (0.871, 1.916) |
|  | **Transplant** | 350 | 0.63 (0.56, 0.71) |  |  | -0.187 (-0.72, 0.341) | 0.832 (0.432, 1.245) |
| **Complete case^1^** | **All** | 370 | 0.73 (0.66, 0.79) | 0.21 | 0.16 | 0 (-0.357, 0.364) | 1 (0.723, 1.295) |
|  | **eGFR<60** | 195 | 0.75 (0.68, 0.83) |  |  | 0.048 (-0.349, 0.461) | 1.221 (0.817, 1.665) |
|  | **eGFR<45** | 84 | 0.77 (0.67, 0.87) |  |  | 0.084 (-0.413, 0.595) | 1.53 (0.72, 2.35) |
|  | **Transplant** | 189 | 0.64 (0.53, 0.74) |  |  | -0.445 (-1.102, 0.194) | 1.31 (0.12, 2.51) |
| **Secondary outcome** | |  |  |  |  |  |  |
| **Complete case^1^** | |  |  |  |  |  |  |
|  | **All** | 567 | 0.72 (0.67, 0.77) | 0.17 | 0.17 | 0 (-0.3, 0.31) | 1 (0.75, 1.27) |
|  | **eGFR<60** | 308 | 0.69 (0.63, 0.75) |  |  | -0.03 (-0.37, 0.31) | 1.03 (0.65, 1.43) |
|  | **eGFR<45** | 147 | 0.7 (0.61, 0.78) |  |  | 0.21 (-0.23, 0.66) | 1.14 (0.58, 1.74) |
|  | **Transplant** | 267 | 0.65 (0.58, 0.72) |  |  | -0.13 (-0.49, 0.24) | 0.8 (0.42, 1.2) |

**Table S3b: Model performance measures for primary and secondary outcomes for validation cohorts**

|  | | **Validation cohort – Ontario, Canada** | | | | | | **Validation cohort –UK** | | | | | |
| --- | --- | --- | --- | --- | --- | --- | --- | --- | --- | --- | --- | --- | --- |
| **Primary outcome** | | **N** | **AUROC (95% CI)** | **R^2^** | **Brier score** | **Calibration intercept** | **Calibration slope** | **N** | **AUROC (95% CI)** | **R^2^** | **Brier score** | **Calibration intercept** | **Calibration slope** |
| **Multiple imputation** | **All** | 6974 | 0.77 (0.72, 0.81) | 0.25 | 0.05 | 0.17 (-0.17, 0.51) | 2.73 (2.46, 3) | 184 | 0.708(0.619 - 0.797) | 0.146 | 0.174 | -0.423 | 0.993 |
|  | **eGFR<60** | 234 | 0.76 (0.69, 0.82) | 0.25 | 0.19 | 0.35 (-0.07, 0.76) | 2.07 (1.4, 2.73) | 116 | 0.711(0.605- 0.817) | 0.172 | 0.191 | -0.452 | 1.267 |
|  | **eGFR<45** | 84 | 0.78 (0.67, 0.88) | 0.27 | 0.21 | 0.36 (-0.16, 0.88) | 2.23 (1.08, 3.38) | 55 | 0.722 (0.583- 0.86) | 0.203 | 0.208 | -0.073 | 1.423 |
|  | **Transplant** | 57 | 0.73 (0.53, 0.93) | 0.15 | 0.13 | 1.05 (-1.25, 3.36) | 1.92 (0.18, 3.66) | 47 | 0.646 ( 0.43- 0.863) | 0.042 | 0.173 | -0.348 | 0.631 |
|  | **ADPKD** | n/a |  |  |  |  |  | n/a |  |  |  |  |  |
|  | **IgAN** | n/a |  |  |  |  |  | n/a |  |  |  |  |  |
| **Complete case^1^** | **All** | 6974 | 0.76 (0.71, 0.8) | 0.22 | 0.03 | -0.47 (-0.76, -0.17) | 1.79 (1.61, 1.98) | 260 | 0.673 (0.595-0.752) | 0.109 | 0.19 | -0.284 | 1.117 |
|  | **eGFR<60** | 234 | 0.75 (0.68, 0.81) | 0.23 | 0.19 | 0.07 (-0.3, 0.43) | 1.38 (0.92, 1.84) | 170 | 0.663 (0.569-0.758) | 0.106 | 0.208 | -0.244 | 1.349 |
|  | **eGFR<45** | 84 | 0.77 (0.66, 0.88) | 0.26 | 0.20 | 0.13 (-0.36, 0.62) | 1.53 (0.72, 2.35) | 84 | 0.656 (0.528-0.784) | 0.115 | 0.223 | 0.013 | 1.419 |
|  | **Transplant** | 57 | 0.72 (0.52, 0.93) | 0.15 | 0.13 | 0.37 (-1.36, 2.09) | 1.31 (0.12, 2.51) | 54 | 0.672 (0.467-0.877) | 0.097 | 0.169 | -0.202 | 1.145 |
| **Secondary outcome** | |  | | | | | | | | | | | |
| **Complete case^1^** | |  |  |  |  |  |  |  |  |  |  |  |  |
|  | **All** | 2144 | 0.77 (0.73, 0.82) | 0.21 | 0.08 | -0.48 (-0.79, -0.17) | 2.03 (1.73, 2.33) | 111 | 0.683 ( 0.565- 0.8) | 0.115 | 0.185 | -0.337 | 1.017 |
|  | **eGFR<60** | 177 | 0.62 (0.54, 0.71) | 0.06 | 0.23 | -0.25 (-0.58, 0.09) | 1.03 (0.29, 1.77) | 61 | 0.671 ( 0.529- 0.813) | 0.093 | 0.22 | -0.181 | 1.093 |
|  | **eGFR<45** | 67 | 0.63 (0.49, 0.77) | 0.06 | 0.24 | -0.16 (-0.68, 0.35) | 1.23 (-0.2, 2.66) | 32 | 0.561( 0.353- 0.768) | -0.009 | 0.251 | -0.054 | 0.424 |
|  | **Transplant** | 48 | 0.68 (0.46, 0.9) | 0.13 | 0.18 | -0.84 (-1.74, 0.06) | 1.5 (-0.07, 3.06) | 184 | 0.708(0.619 - 0.797) | 0.146 | 0.174 | -0.423 | 0.993 |

**Table S4: Characteristics of women in development cohort stratified by the primary and secondary outcome**

|  |  | Primary outcome | | | | Secondary outcome | | | |
| --- | --- | --- | --- | --- | --- | --- | --- | --- | --- |
| Variable |  | <25% | =>25% | *p* | N with data | No | Preterm < 34 and/or SGA 3^rd^ centile | *p* | N with data |
| N (%) |  | 525 (70.4%) | 221 (29.6%) |  |  | 422 | 151 |  |  |
| Age at conception (mean (SD)) |  | 32.3 (5) | 30.9 (5.1) | 0.0004 | 746 | 32.2 (4.9) | 31.3 (5.3) | 0.084 | 571 |
| Ethnicity (%) | Asian | 65.6 | 34.4 | 0.36 | 742 | 71.2 | 28.8 | 0.37 | 568 |
|  | Black | 60.0 | 40.0 |  |  | 90.0 | 10.0 |  |  |
|  | Other | 77.3 | 22.7 |  |  | 76.5 | 23.5 |  |  |
|  | White | 71.2 | 28.8 |  |  | 72.9 | 27.1 |  |  |
| Multiparous (%) | Nulliparous | 72.7 | 27.3 | 0.20 | 738 | 66.4 | 33.6 | 0.0004 | 567 |
|  | Multiparous | 68.3 | 31.7 |  |  | 79.5 | 20.5 |  |  |
| Proteinuria (%) | Normal | 84.0 | 16.0 | <0.0001 | 370 | 78.5 | 21.5 | 0.162 | 292 |
|  | Moderate | 79.6 | 20.4 |  |  | 79.0 | 21.0 |  |  |
|  | Severe | 55.6 | 44.4 |  |  | 68.4 | 31.6 |  |  |
| Body Mass Index (median (IQR)) |  | 25.7 (22.6,29.2) | 26.3 (22.7,30.3) | 0.66 | 175 | 26.3 (22.9,30.2) | 24.9 (20.9,27.9) | 0.029 | 145 |
| Chronic Hypertension (%) | No | 70.9 | 29.1 | 0.80 | 746 | 79.0 | 21.0 | 0.017 | 571 |
|  | Yes | 70.0 | 30.0 |  |  | 70.0 | 30.0 |  |  |
| Diabetes (%) | No | 70.9 | 29.1 | 0.37 | 746 | 75.8 | 24.2 | 0.0001 | 571 |
|  | Yes | 65.7 | 34.3 |  |  | 51.9 | 48.1 |  |  |
| Pre pregnancy eGFR (median (IQR)) |  | 60.2 (47.4,74) | 50.6 (36.5,64.6) | <0.0001 | 746 | 60.1 (48.0,73.3) | 49.2 (35.2,65.7) | <0.0001 | 571 |
| CKD Cause (%) | ADPKD | 62.4 | 37.7 | 0.004 | 721 | 85.7 | 14.34 | 0.018 | 549 |
|  | Chronic Pyelonephritis or Vesicoureteral reflux | 62.5 | 37.5 |  |  | 71.7 | 28.3 |  |  |
|  | Congenital/Inherited | 68.2 | 31.8 |  |  | 71.4 | 28.6 |  |  |
|  | Diabetic Nephropathy | 36.4 | 63.6 |  |  | 50.0 | 50.0 |  |  |
|  | Glomerulonephritis | 63.9 | 36.1 |  |  | 80.2 | 19.8 |  |  |
|  | SLE | 70.0 | 30.0 |  |  | 75.0 | 25.0 |  |  |
|  | Other | 62.9 | 37.1 |  |  | 82.1 | 17.9 |  |  |
|  | Transplant | 76.9 | 23.1 |  |  | 66.5 | 33.5 |  |  |
| Diabetic nephropathy^1^ (%) | Other PRD | 69.9 | 30.1 | 0.38 | 719 | 74.1 | 25.9 | 0.002 | 548 |
|  | Diabetic nephropathy | 63.2 | 36.8 |  |  | 48.4 | 51.6 |  |  |
| Postpartum eGFR (median (IQR)) |  | 56.8 (44.7,71.7) | 26.6 (20.6,41.6) | <0.0001 | 728 | 52.7 (39.6,69.6) | 37.6 (24.4,54.3) | <0.0001 | 556 |
| Gestation weeks (median (IQR)) |  | 37 (35,38) | 35 (32,37) | <0.0001 | 578 | 37 (36,38) | 32 (29,33) | <0.0001 | 612 |
| Delivery mode (%) | Elective CS | 62.2 | 37.8 | 0.004 | 746 | 77.2 | 22.8 | <0.0001 | 571 |
|  | Emergency CS | 70.6 | 29.4 |  |  | 56.8 | 43.2 |  |  |
|  | Instrumental/breech | 83.3 | 16.7 |  |  | 85.4 | 14.6 |  |  |
|  | Spontaneous vaginal | 73.8 | 26.2 |  |  | 85.6 | 14.5 |  |  |
| Livebirth (%) | Livebirth | 71.2 | 28.8 | 0.012 | 633 | 74.1 | 25.9 | 0.003 | 567 |
|  | Stillbirth | 20.0 | 80.0 |  |  | 0.0 | 100.0 |  |  |
| Birthweight (g) (median (IQR)) |  | 2775 (2230,3160) | 2300 (1770,2792) | <0.0001 | 631 | 2863 (2480,3210) | 1680(1260,1960) | <0.0001 | 565 |
| Sex of baby (%) | Male | 72.2 | 27.8 | 0.25 | 616 | 75.7 | 24.3 | 0.16 | 543 |
|  | Female | 68.0 | 32.0 |  |  | 70.4 | 29.6 |  |  |

1 Includes transplant patients with diabetic nephropathy as PRD, who are included under “Transplant” in “CKD Cause”.


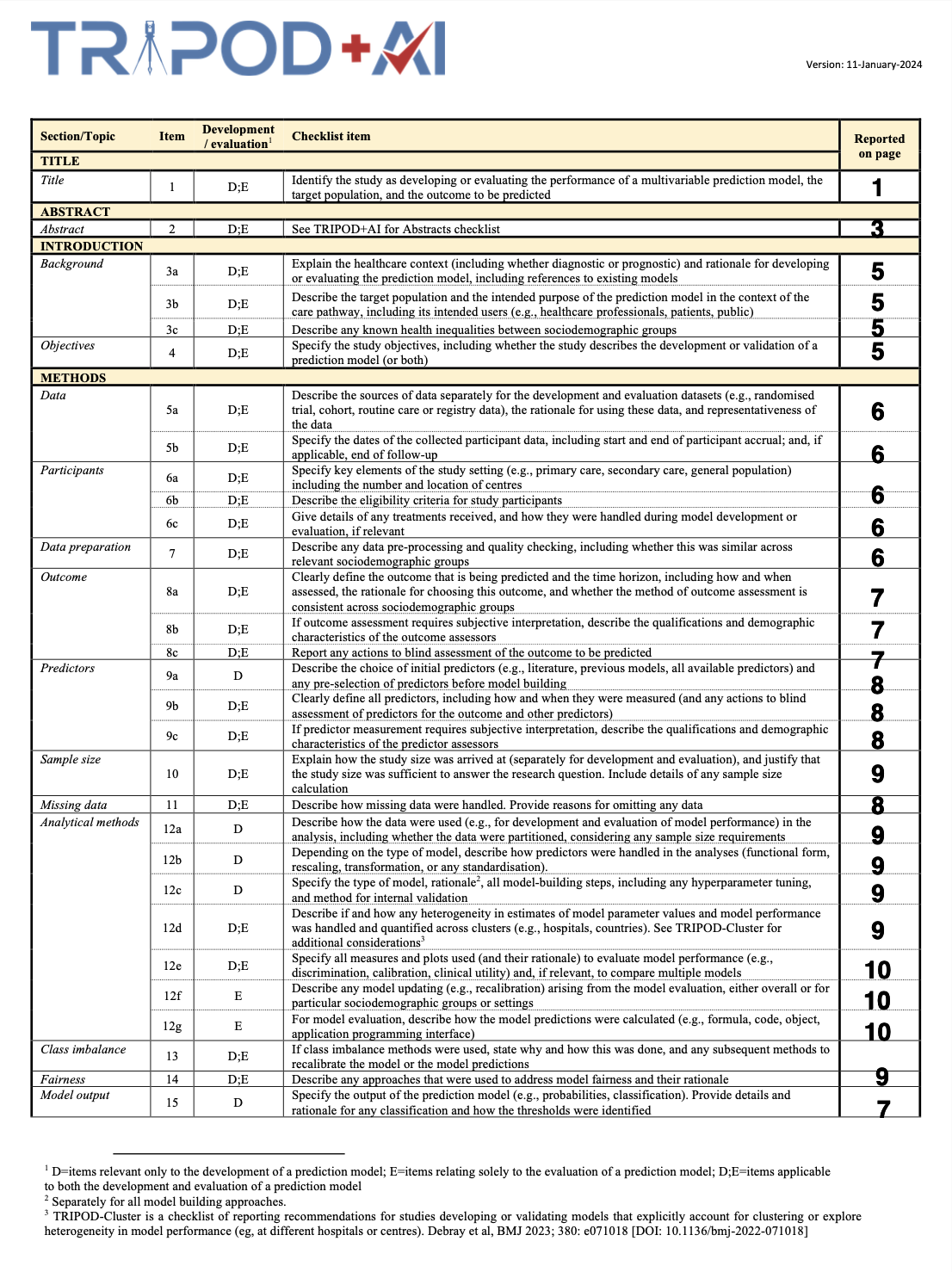


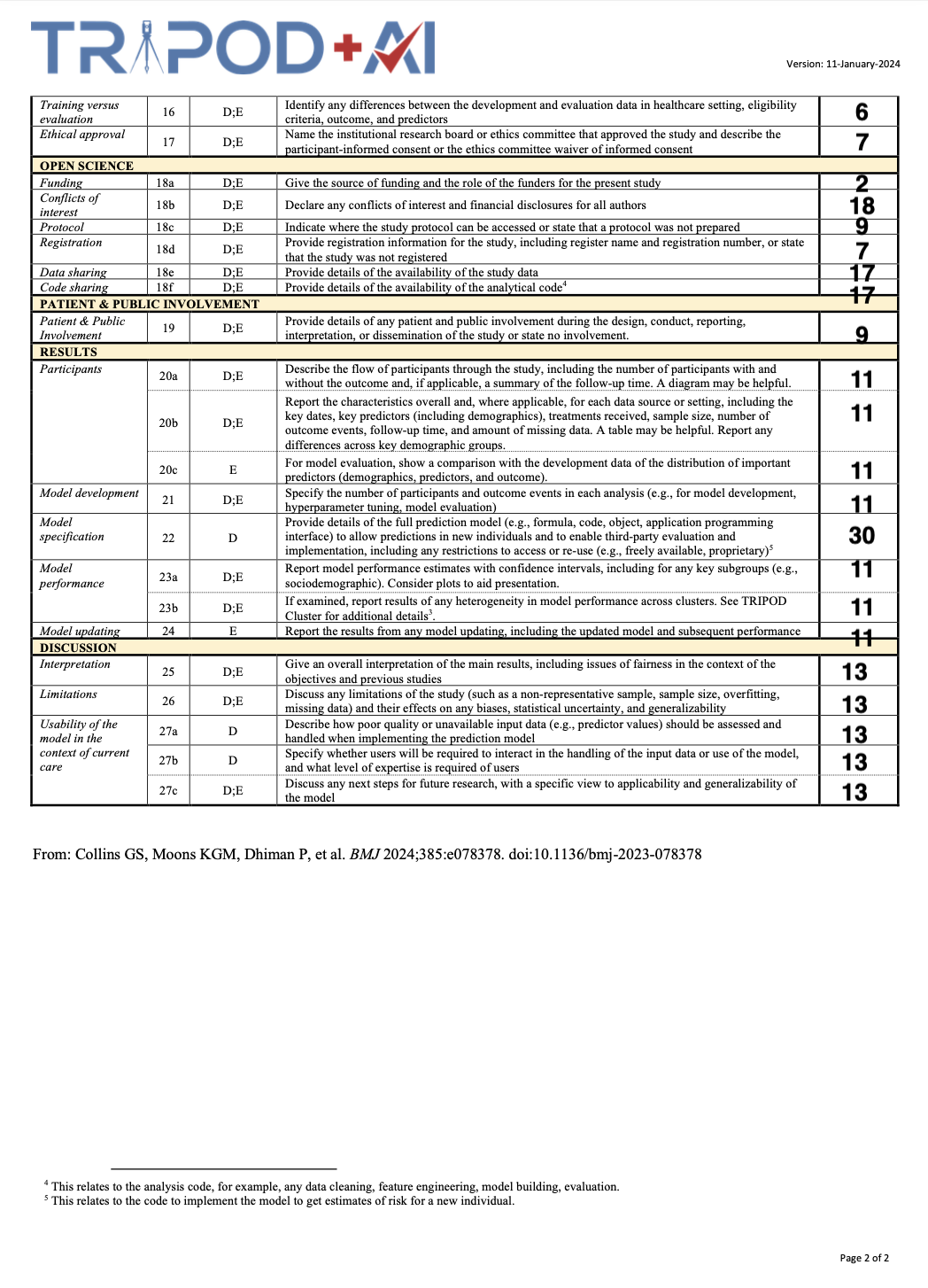

Supplement: Supplementary File (PDF) — Table S1. Missing data within the overall PREDICT cohort. Table S2. Prediction model parameters from multivariable regression. Table S3. Model performance measures for primary and secondary outcomes. Table S4. Characteristics of women stratified by the primary and secondary outcome. TRIPOD checklist. [file mmc1.docx]
